# Psychosocial Aspects for Patients and Family Members

**DOI:** 10.1080/135771402753667727

**Published:** 2002-05


### PSYCHOSOCIAL ASPECTS FOR PATIENTS AND FAMILY MEMBERS

### ORAL PRESENTATIONS

### I134 The Role of Lymphedema Treatment in the Rehabilitation after Limb-sparing Surgery

I. Meller, R. Oren, J. Bickels, A. Rassad, Y. Kollender
*Tel Aviv Sourasky Medical Center, Tel Aviv, Israel*

*Limb-sparing surgeries may be associated with significant injury to the soft-tissues, compromised lymph-drainage, and lymphedema. The current study summarizes our experience with decongestive-lymphatic-therapy (DLT) in those patients. *Between 1997 and 2001, 26 patients were referred to the DLT-service. There were 15 females and 11 males (age 19–73 years) and diagnosed with 20 soft-tissue and 6 bone tumors. 21 tumors were located in the lower extremities (thighs – 16; legs – 5) and the remaining 5 in the upper extremities (arms – 4; forearms – 2). Nine patients underwent one limb-sparing surgery, 11 patients underwent 2–3 surgeries, and 6 patients underwent more than 4 surgeries. 24 patients were treated with adjuvant radiation therapy. A correlation was noted between the number of surgeries, a proximal anatomic location (thigh/arm), and the magnitude of lymphedema. At the time of referral, all patients had a significant swelling of the operated extremity and associated loss of cosmesis, 21 reported functional limitation, and 2 reported pain. A DLT-protocol consisted of 24 sessions, given over a period of 5 weeks. Each session entailed skin care, manual lymph drainage, bandaging, and active exercises. *Improvements in the volume and cosmesis of the treated extremity were estimated to be good or excellent in 14 patients, moderate in 9 patients, and minimal in 3 patients. 17 of 21 patients with functional limitation and 1 of 2 patients with pain reported significant improvement. Patients who were referred early to DLT typically had a better outcome that was achieved in shorter period. *DLT can improve swelling, loss of cosmesis, functional deficit, and pain associated with limb-sparing surgery. Multiple surgeries, adjuvant radiation therapy and proximal anatomic location are risk factors to the evolution of lymphedema. Recognition of these risk factors and early administration of treatment may achieve even greater improvement in these patients.

### I135 The Quality of Interaction between the Operators, The Oncological Patient and His Family

F. Tanganelli, A. Corsi, V. Delfino
*Orthopedic and Traumatologic Center, Florence, Italy*

The nurse culture has always treated the question of global approach to the suffering person, and so also of the needs tied to his private, emotional and relational field. This is even more true in oncology because the event of cancer summons pictures of degeneration, suffering and loneliness. In oncology one does not need only to be a good nurse or doctor but it's must that these operators accept the sick person as a whole of: personal experiences, reaction to the diagnosis, capacity to reorganize one's life. Going straight to the point the operators must have the capacity to show 'competence in communicating', a competence that will allow them to successfully solve the problem of relationship with the patient and his familiars, the problem of communicational integration with the collegues and the problem on how to handle personal emotional reactions and also one's personal experience in front of patological situation in which there is a strong proximity with death.

### I136 Project for a School Service

E. Sgroi, F. Forni, G. Bacci
*Istituti Ortopedici Rizzoli, Bologna, Italy*

A project for a school service for teen-agers patients at the Department of Musculoskeletal Oncology of Istituto Ortopedica Rizzoli started in 1997, after a research showing the majority of these patients to loose their classes, but also their school-friends, because of physical impairment caused by chemotherapy and surgery employed to treat their bone sarcomas. Besides the teaching/learning purposes, this project aimed to give these young patients something to 'think about', in order not to focus on illness and fears, and trying to keep the fundamental goals of a normal mental and physical development central for the life of these people and their families too. The project involves young patients from all over Italy, attending all kinds of schools, who are individually followed by a large group of qualified teachers for all the subjects required by their school-curricula. The teachers and a coordinator are in touch with their schools, to assure a continuing contact between these boys and girls and their original background. Since 1997, 150 patients aged between 11 and 19 years followed this iniative and more than 10.00 hours of lessons were given together with credits and official evaluations. The interaction between teachers and the Musculoskeletal Oncology Dept. staff was optimal, since treatments and cures were re-scheduled, whenever possible, to respect the time of lessons and patients' needs. This initiative gave very important results, since it produced a much better attitude and acceptance of hospitalization. Moreover, the collaboration between the staff, the teachers, and the families was a strong and real support for patients for their positive mental and physical development.

### I137 The Experience of having Cancer

J. Woodford
*Royal Nat. Orthop. Hospital NHS Trust, Middlesex, United Kingdom*

*Introduction:* Cancer is experienced as a complex, multifaceted illness rather than a disease process. Cancer nurses have a responsibility to seek and interpret such lived experiences to ensure therapeutic care is delivered.
*Aim of the Study:* The aim of the study was to investigate the contribution of phenomenology as a research paradigm that enables nurses to discover, interpret and understand the experiences of those with cancer.
*Method:* Criteria for evaluating qualitative research that examines the credibility, transferability, dependability and confirmability were employed to establish the trustworthiness of the studies.
*Results:* Analysis of the studies with supporting literature identified four themes that existed within a concurrent concept of transition. These were contextually related to the cancer trajectory and included cancer as life changing through an existential awareness, the personal endeavour to maintain control and balance, a search for inner meaning and the importance of family support throughout. Sub themes identified included living with uncertainty, encountering personal growth and fulfilment and a desire to help others.
*Conclusions:* The findings of this study are directly relevant within today's healthcare culture where there appears to be an increasing emphasis on the scientific and technical advancements of cancer diagnosis and treatment. The experience of having cancer is confirmed as complex and highly individualised requiring dynamic and therapeutic nursing support. The appropriateness of phenomenology is confirmed as an effective research method and will be used in a future study to explore the lived experience of having limb salvage surgery for sarcoma.

**I138 The Relation between Osteosarco MA, Skiing and Holidays for Kids**

N. Donders
*SKOV, Almere, The Netherlands*

In 1985 the Foundation for Children Oncology Holiday Camps (SKOV) was established by Cees Donkervoort. His mission was to organise a sailing camp for childeren suffering cancer in order te give them a carefree holiday with medical attention. That the parents of those children would be free of care, during that week, for their sick child was seen as a added advantage. The foundation started with one camp 'Summerfolly' hosting 25 young people. At this moment the foundation organises 5 camps annually hosting 450 sick children. During a one day camp brothers, sisters and parents are allowed to participate in the camp. This extra group is for about 750 people. All camps are organised, served and guided by volunteers. Most of them are medically trained professionals. The annual costs of the camps amount to about Ä 200.000. These costs are covered by financial return on the capital plus annual sponsoring of Ä 50.000. The capital and sponsoring is raised by private initiative like, schools, service clubs and enterprises. As an ex-patient who suffered an upperlegamputation due to an osteosarcoma the author will share her experience about the changes in her life caused by the surgical intervention and how life went on. The relationship between the speaker, skiing and the SKOV will be discussed. She helped to establish one of the wintercamps 'winternonsense' to which special attention will be given.

### POSTER PRESENTATIONS

**I139 The Assessment of Comorbidity in Sarcoma Patients: How Useful in Treatment and Outcome?**

L.C. Russell, A. Hughes, A. Kulkarni, R.J. Grimer
*Royal Orthopaedic Hospital, Birmingham, United Kingdom*

*Aim:* Patients with soft tissue sarcomas often have other medical conditions (eg. cardiac, vascular, neurological etc) which may affect their treatment and may also be more relevant to prognosis than their actual tumour. This study investigates a simple tool – ACE 27 – for assessing comorbidity and incorporating that into a prognostic algorithm.
*Method:* The ACE 27 is implemented through a questionnaire which focuses on all the body systems individually to ensure that any co-existing conditions are detected. The questionnaire will be implemented prior to there being a definitive diagnosis and will run alongside the rapid assessment questionnaire. On completion of the tool the patients will be given a score which indicates the degree of severity of any other medical conditions. Comparison with an age matched population without sarcomas will be used to assess the relative health of the two groups.
*Results:* The presentation will detail the ease with which this information can be collected and stored and its use in managing patients during their hospital stay. For prognostic purposes a retrospective review of notes has also been used to assess ACE 27 scores and this has been used to factor into a prognostic scoring system for STS.
*Conclusion:* Comorbidity is a much underestimated problem in older patients with sarcomas. We have shown that a simple scoring system is of help not only in treating patients but also in assessing outcome.

**I140 The Correlation between Response to I.V. Strontium 89 and the Response to External Beam Radiation to Limited Field in the Palliation of Bone Pain in Patients with Osteoblastic Bone Metastases**

Z. Shmuely, I.G. Ron, O. Stav, F. Kovner
*Tel Aviv Sourasky Medical Center, Tel Aviv, Israel*

Forty-three cancer patients with skeletal pain due to osteoblastic metastases were the population of the research. The patients were treated with hormonal or chemotherapy according to their primary malignancies, and were treated with analgetic pharmacotherapy as needed. Most patients(N=36) received local external beam radiotherapy as a palliative treatment. The efficacy of local external radiation in metastatic bone pain palliation was found to be high (80.6%). Results suggest that response to external radiotherapy may be viewed as a prognostic indicator of Strontium-89 efficacy in metastatic osteoblastic bone pain palliation in the same patient. It has been assumed that under these research conditions the 'duration of response' criterion is biased towards a shorter response after Strontium therapy, due to its late administration in the course of the disease and the chosen line of treatment. This assumption is favored by the relatively high efficacy of Strontium (71.4%) amongst the 7 patients who received Strontium-89 without previous radiation therapy (Strontium-89 used in early stage of bone metastases).

**I141 Functional Recovery of the Patellofemoral Joint after Resection and Total Knee Arthroplasty due to Distal Femoral Osteosarcoma**

A. Cotti[1], C Goretti[1], O. Marchese[1], M. Monica[2]
*[1]Istituti Ortopedici Rizzoli, Bologna, Italy, [2]Mayer Institute, Florence, Italy*

Correct patellofemoral joint function is essential for good function of the knee joint. This joint must be taken into account when performing total knee arthroplasty in patients with distal femoral osteosarcoma. A detailed analysis of the clinical picture enables a good plan of recovery to be formulated so that our treatment can be aimed at reasonably achievable objectives. Certain factors influence joint recovery after total knee arthroplasty due to distal femoral osteosarcoma:

- The amount of bone resected;
- The amount of muscle removed;
- Type of prosthesis and operative technique used;
- Alignment of the patella after surgery.

Rehabilitation, which plays a fundamental role in the recovery of these patients, must also take into consideration the new joint between the prosthesis and the patella. Passive exercises of this joint and detachment of scar and adjacent tissue enable periarticular adherence to be restricted. Thus, with rehabilitation we can aim at better joint recovery and therefore a better final functional result. The best results are obtained by a detailed examination of all clinical pictures and by adapting the rehabilitation program to them.

